# COVID-19 Pandemic: Knowledge, Attitude, and Perception of Medical Students Toward the Novel Coronavirus Disease

**DOI:** 10.1017/dmp.2021.169

**Published:** 2021-06-07

**Authors:** Hesham Elsayed Emara, Abdallah Ahmoud Alhindi, Hisham Ahmed Orebi, Ibrahim Ali Kabbash, Noha M. Elghazally

**Affiliations:** 1 Faculty of Medicine, University of Tanta, Egypt; 2 Public Health & Community Medicine, Faculty of Medicine, University of Tanta, Egypt

**Keywords:** COVID-19, knowledge, medical students, attitude, prevention

## Abstract

**Background:**

Medical students are vulnerable to infection by the coronavirus. Their awareness of the disease is crucial for their safety and for the management of the epidemic by spreading supportive information in their communities. The aim of this study was to assess coronavirus disease 2019 (COVID-19)-related knowledge, attitude, and preventive practices among Egyptian medical students.

**Methods:**

We conducted a cross-sectional study from the beginning of April to June 2020; a total of 439 undergraduate medical students (1st to 6th academic years) were assessed using an online questionnaire. The questionnaire consisted of 33 questions, including 5 items regarding socio-demographic features, 23 items concerning COVID-19 related knowledge, 2 items regarding attitude, and 3 items related to preventive measures.

**Results:**

We observed an acceptable level of knowledge (74.3%) among the sample studied. Preclinical and female students were significantly more optimistic as 69.1% expected successful control of COVID-19, and 48.9% predicted that Egypt will win the fight against COVID-19. The majority of participants reported wearing a facemask in public places as a preventive measure (56.7%).

**Conclusions:**

Egyptian medical students had an acceptable level of knowledge, positive attitude, and good practices of preventive measures regarding the COVID-19 virus. There is no significant difference in almost all items of knowledge, attitude, and practices in relation to gender or academic grade.

Coronavirus disease 2019 (COVID-19) is an evolving contagious respiratory disease caused by a new coronavirus. China first identified the virus, in December 2019, and infection started to spread rapidly in China and many other countries to represent a global health crisis.^1^ The main clinical symptoms of COVID-19 include fever, fatigue, dry cough, myalgia, and shortness of breath. The International Committee on Taxonomy of Viruses (ICTV) named the virus severe acute respiratory syndrome coronavirus 2 (SARS-CoV-2).^2^

Egypt identified its first COVID-19 case on February 14, 2020. According to daily reported statistics of the Ministry of Health and Population in Egypt, the total confirmed cases have reached 100,557 by September 10, 2020, with total deaths of 5590 in Egypt.^3,4^ The World Health Organization (WHO) declared the novel coronavirus a public health emergency in response to the alarming situation and rapidly increasing number of cases all over the world. The WHO warned all countries to undertake emergency preparedness measures to face the current situation through many means, including active surveillance, early detection, and isolation.^5^

Although medical education has been affected because of the wide spread of the COVID-19 pandemic, opinions differ regarding the convenient participation of medical students in clinical care among different institutions.^6^ However, medical students had a salient role in care of patients during the Spanish flu pandemic in 1918 and the polio epidemic in 1952 in Denmark.^7,8^

The Egyptian government adopted preventive measures to control COVID-19 spread in all governorates. These measures included self-isolation at home; closure of public places, such as shopping centers, restaurants, sport clubs; and isolation of suspected cases and infected patients. In addition, schools and universities were closed.^9^ Moreover, despite suspending medical education in Egypt for many weeks, authorities advocated streamed online lectures to keep students on track with teaching.^10^

We conducted the current study to assess knowledge, attitudes, and practices regarding COVID-19 among medical students at Tanta University, Faculty of Medicine, and to detect any gender and academic grade difference among participants.

## Methods

### Study Design and Settings

A cross-sectional study was conducted at the Faculty of Medicine, Tanta University, during the period from April 1 to June 30, 2020.

### Participants

The study population was undergraduate medical students (1st to 6th academic years). The total number of the study population was 5340. We calculated the sample size using Epi Info 7 software. We assumed the probability of having good knowledge and a positive attitude toward preventive measures against coronavirus disease at 50.0 %,^11^ with 97% confidence interval and 5% precision. The calculated sample size was 434 participants. We recruited a convenient sample of 439 students.

### Study Tool

The authors designed a self-structured questionnaire sheet to collect data based on the survey instrument developed in a study on Chinese residents’ knowledge, attitudes, and practices (KAP) toward COVID-19 in China.^12^ The questionnaire was comprised of 3 sections with 33 questions as follows:

#### Section I

This section included 5 items centered on students’ characteristics: age, gender, year of study, residence, and last year’s achievement grade.

#### Section II

This section measured knowledge regarding COVID-19. Thirteen questions were adapted from previous research, and authors added another 10 questions. The 23 items assessing knowledge were answered by “true,” “false,” or “do not know” options. Other questions had different response options (items 14, 18, 23). We scored right answers by a 1 point, whereas a wrong/not sure answer was assigned 0 points. Thus, a total score ≥ 70% (16 points and more) was designated as acceptable, 50% to <70% (12-15 points) as moderate, and less than 50% (11 points and less) as low level of knowledge.

#### Section III

This section measured attitudes and practices toward COVID-19, including 5 questions. Two questions regarding the students’ attitude: if they expected successful control of the current pandemic, and their trust in the Egyptian authorities to win the fight against COVID-19. Students had to answer with agree, disagree, or not sure. Three questions measured practices with yes/no answers: if participants tried to avoid going to overcrowded places, wore a mask when going outside the home, and if they cleaned their houses with disinfectants.

### Validity of the Tool

Two consultants of infectious diseases and 3 Egyptian professors from the epidemiology department assessed validity of the questionnaire. The panel of experts reviewed both the website and the questionnaire. For the website, experts provided helpful techniques about general use by participants. For the questionnaire, reviewers recommended shortening of some questions and proposed minor changes in 4 knowledge section questions (questions (2, 5, 8, and 9). Regarding the time required to finish the questionnaire by participants, experts stated that all questions were understandable and participants can fill it out in 7 to 10 min.

We tested the questionnaire in a pilot study to assess its reliability by recruiting 20 medical students not included in current study. We used data to assess internal consistency using alpha Cronbach and test-retest reliability by using the intra-class correlation coefficient, (with Cronbach’s alpha = 0.82 and the intra-class correlation coefficient was 0.91), which represented adequate internal consistency and reliability.

### Data Collection

We collected data online through Google Forms posted on the Internet platforms. We posted the questionnaire on social media platforms, and shared it on Facebook groups of Tanta medical students. Responders were 439 students who shared in the study voluntary and anonymously.

Data were stored on a network-attached storage solution, the cloud, which made them available through a network connection. Once the data were collected, researchers developed a data-sharing scheme with a secure password to guarantee confidentiality of data owners and the security of the outsourced cloud data, and securely destroying the data after completing the research.

### Statistical Analysis

We used SPSS version 20 (Statistical Package for Social Studies) created by IBM, Chicago, Illinois, to organize and tabulate the collected data. We used descriptive statistical methods to calculate numbers and percentages for each variable. The chi-squared test was used to assess potential statistical relationships between socio-demographic and knowledge, attitudes, and precautionary measures. If chi-squared was inappropriate, we used the Fisher exact test. We adopted the value of *P* < 0.05 for statistical significance.

### Ethical Considerations

The Ethical Committee of Scientific Research in Tanta Faculty of Medicine approved the research before starting the study. For participant consent, we inserted a written consent in the introductory part of the online survey; all participants consented before answering the questions.

## Results

### Demographic Characteristics

The total number of participants was 439. Table 1 shows demographic characteristics of participants; females represented 52.6%. Approximately half of the participants were 22 to 23 y old (47.4%), one-third of them were between 18 and 19 y (29.8%), whereas only 8.2% were 24 y and older and 14.6% were 20 and 21 y. Most of the participants (43.1%) were 5th year medical students, and one-quarter of them were 1st year students (24.8%). Nearly half of the participants (47.2%) had excellent grades in their last year of university. More than half of the students (55.4%) lived in urban areas.

**Table 1.** Characteristics of studied medical students in relation to gender

Variables	Males (*N* = 208)		Females (*N* = 231)		Total (*N* = 439)		*t/X* ^*2*^	*P*-Value
	*n*	%	*n*	%	*n*	%		
Age (y)							1.509	0.132
Range	18-25		18-25		18-25			
Mean + SD	21.49+2.00		21.21+1.86		21.49+2.00			
Current academic year								
Preclinical years	92	44.2	102	44.2	194	44.1	0.001	0.987
Clinical years	116	55.7	129	55.9	245	55.9		
Achievement last year							13.754	0.003*
Excellent	86	41.3	121	52.4	207	47.2		
Very good	70	33.7	76	32.9	146	33.3		
Good	39	18.8	32	13.9	71	16.2		
Satisfactory	13	6.3	2	0.9	15	3.4		
Residence							0.875	0.350
Urban	120	57.7	123	53.2	243	53.2		
Rural	88	42.3	108	46.8	196	44.6		

* *P* < 0.05.

### Assessment of Knowledge

The average knowledge score for students was 17.1 ± 1.9 (range = 0-23). The overall correct answer rate of the knowledge questionnaire was 74.3%, and the ranges of correct answer rates for all students were 31.9% to 94.8%. Undergraduates who had scores above 17 were 57.2% (*n* = 251), indicating an acceptable level of knowledge on COVID-19.

Knowledge of infection of COVID-19 virus by droplets from infected patients was reported by 98.6%; prevention of COVID-19 infection requires people to avoid visiting overcrowded places and avoiding taking public transportation was reported by 97.5%. The majority of participants had an awareness of the major clinical symptoms of COVID-19 (76.6%). Only 47.2% of participants gave a correct answer when asked if eating or contacting wild animals may cause infection by the COVID-19 virus. Among participants, 11.8% believed that it is not necessary for juniors and youngsters to follow the preventive measures against being infected by COVID-19 virus (Table 2).

**Table 2.** Comparison of knowledge about COVID-19 among medical students in relation to gender

Items of knowledge of COVID-19	Males (*N* = 208)		Females (*N* = 231)		Total (*N* = 439)		*X* ^*2*^	*P*-Value
	*n*	%	*n*	%	*n*	%		
Main clinical symptom								
Fever	190	91.3	224	97.0	414	94.3	6.445	0.011*
Fatigue	153	73.6	191	82.7	344	78.4	5.376	0.020*
Dry cough	196	94.2	223	96.5	419	95.4	1.339	0.247
Dyspnea	157	75.5	180	77.9	337	76.8	0.366	0.454
Myalgia	78	37.5	60	39.0	168	38.3	0.099	0.753
Unlike common cold, stuffy nose, runny nose, and sneezing are less common	147	70.7	171	74.0	318	72.4	0.616	0.432
Currently, there is no effective cure	180	86.5	193	83.5	373	85.0	0.765	0.382
Early symptomatic and supportive treatment can help most patients recover from the infection	179	86.1	204	88.3	383	87.2	0.500	0.480
Low-risk groups								
Children	114	54.8	127	55.0	241	54.9	0.001	0.971
Adults	175	84.1	176	76.2	351	80.0	4.310	0.038*
High-risk groups								
Pregnant females	115	55.3	162	70.1	277	63.1	10.35	0.001*
Elderly people	193	92.8	220	95.2	413	94.1	1.179	0.278
Obese patients	89	42.8	91	39.4	180	41.0	0.521	0.470
Chronic patients	176	84.6	213	92.2	389	88.6	6.251	0.012*
Eating or contacting wild animals would not result in infection	100	48.1	107	46.3	207	47.2	0.136	0.713
Infected persons can transmit the virus to others when fever is not present	176	84.6	192	83.1	368	83.8	0.181	0.670
COVID-19 virus spreads via respiratory droplets of infected individuals	208	100.0	225	97.4	433	98.6	FE	0.031*
People can wear general medical masks to prevent infection	143	68.8	154	66.7	297	67.7	0.217	0.641
It is necessary for children and young adults to take measures to prevent infection	185	88.9	202	87.4	387	88.2	0.235	0.628
To prevent infection individuals should avoid going to crowded places and avoid taking public transportations	203	97.6	225	97.4	428	97.5	0.017	0.897
Isolation and treatment of people who are infected are effective to reduce spread of the virus	202	97.1	216	93.5	418	95.2	3.130	0.077
People who have contact with someone infected virus should be immediately isolated in a proper place	191	91.8	217	93.9	408	92.9	0.744	0.388
The observation period for people who have contact with patients should be 7-14 days	122	58.7	131	56.7	253	57.6	0.169	0.681
The virus mainly affects lungs, and can cause permanent lung damage	182	87.5	196	84.8	378	86.1	0.643	0.423
The virus can live on surfaces for a long period of time	129	62.0	138	59.7	267	60.8	0.239	0.625
The virus can be transmitted in a hot climate	118	56.7	120	51.9	238	54.2	1.009	0.315
Diagnosis can be confirmed by PCR	182	87.5	198	85.7	380	86.6	0.300	0.584
The flu vaccine cannot protect against COVID-19 virus	141	67.8	161	69.7	302	68.8	0.186	0.667
The incubation period of COVID-19 virus is 2-14 days	177	85.1	186	80.5	363	82.7	1.601	0.203
Older people who suffer from chronic medical conditions are more vulnerable to becoming ill with COVID-19 virus	191	91.8	206	89.2	397	90.4	0.888	0.346
Plasma of cured COVID-19 patients can be effective in treatment of infection	110	52.9	122	52.8	232	52.8	0.001	0.988
Most common ethnic group at high risk of mortality if infected are black persons	22	10.6	14	6.1	36	8.2	2.966	0.085

* *P* < 0.05.

**Table 3.** Comparison of attitude and practices about COVID-19 among medical students in relation to gender

Variables	Males (*N* = 208)		Females (*N* = 231)		Total (*N* = 439)		*X* ^*2*^	*P*-Value
	*n*	%	*n*	%	*n*	%		
Do you believe that COVID-19 will finally be successfully controlled?							0.550	0.759
Agree	131	63.0	151	65.4	282	64.2		
Disagree	34	16.3	32	13.9	66	15.0		
Don’t know	43	20.8	48	20.8	91	20.7		
Do you have confidence that Egypt can win the battle against the COVID-19 virus?							10.463	0.005*
Agree	79	38.0	113	48.9	192	43.7		
Disagree	85	40.9	61	26.4	146	33.3		
Don’t know	44	21.2	57	24.7	101	23.0		
In recent days, have you gone to any crowded place?							0.006	0.941
Yes	48	23.1	54	23.4	102	23.2		
No	160	76.9	177	76.6	337	76.8		
In recent days, have you worn a mask when leaving home?							0.922	0.337
Yes	113	54.3	136	58.9	249	56.7		
No	95	45.7	95	41.1	190	43.3		
In recent days, have you used disinfectants to clean your house?							0.034	0.853
Yes	198	95.2	219	94.8	417	95.0		
No	10	4.8	12	5.2	22	5.0		

* *P* < 0.05

**Table 4.** Comparison of knowledge about COVID-19 among medical students in relation to academic grade

Items of knowledge of COVID-19	Preclinical (*N* = 194)		Clinical (*N* = 245)		*X* ^*2*^	*P*-Value
	*n*	%	*n*	%		
Main clinical symptom						
Fever	177	91.2	237	96.7	6.093	0.014*
Fatigue	150	77.3	194	79.2	0.222	0.638
Dry cough	184	94.8	235	95.9	0.287	0.592
Dyspnea	148	76.3	189	77.1	0.044	0.833
Myalgia	68	35.1	100	40.8	1.523	0.217
Unlike common cold, stuffy nose, runny nose, and sneezing are less common	133	68.6	185	75.5	2.622	0.105
Currently, there is no effective cure	165	85.1	208	84.9	0.002	0.964
Early symptomatic and supportive treatment can help most patients recover from the infection	174	89.7	209	85.3	1.870	0.171
Low-risk groups for infection						
Children	103	53.1	138	56.3	0.457	0.499
Adults	154	79.4	197	80.4	0.071	0.790
High-risk groups for infection						
Pregnant females	114	58.8	163	66.5	2.806	0.094
Elderly people	181	93.3	232	94.7	0.378	0.539
Obese patients	79	40.7	101	41.2	0.011	0.915
Chronic patients	170	87.6	219	89.4	0.332	0.565
Eating or contacting wild animals would not result in infection	91	46.9	116	47.3	0.008	0.927
Infected persons can transmit the virus to others when fever is not present	168	86.6	200	81.6	1.696	0.161
COVID-19 virus spreads via respiratory droplets of infected individuals	193	99.5	240	98.0	FE	0.235
People can wear general medical masks to prevent infection	123	63.4	174	71.0	2.872	0.090
It is necessary for children and young adults to take measures to prevent infection	165	85.1	222	90.6	3.206	0.073
To prevent infection individuals should avoid going to crowded places and avoid taking public transportations	190	97.9	238	97.1	FE	0.762
Isolation and treatment of people who are infected are effective to reduce spread of the virus	185	95.4	233	95.1	0.016	0.900
People who have contact with someone infected virus should be immediately isolated in a proper place	184	94.8	224	91.4	1.926	0.165
The observation period for people who have contact with patients should be 7-14 days	116	59.8	137	55.9	0.666	0.414
The virus mainly affects lungs, and can cause permanent lung damage	167	86.1	211	86.1	0.001	0.990
The virus can live on surfaces for a long period of time	119	61.3	148	60.4	0.039	0.843
The virus can be transmitted in a hot climate	102	52.6	136	55.5	0.375	0.540
Diagnosis can be confirmed by PCR	167	86.1	213	86.9	0.068	0.794
The flu vaccine cannot protect against COVID-19 virus	123	63.4	179	73.1	4.705	0.030*
The incubation period of COVID-19 virus is 2-14 days	160	82.5	203	82.9	0.011	0.916
Older people who suffer from chronic medical conditions are more vulnerable to becoming ill with COVID-19 virus	173	89.2	224	91.4	0.635	0.425
Plasma of cured COVID-19 patients can be effective in treatment of infection	104	53.6	128	52.2	0.081	0.776
Most common ethnic group at high risk of mortality if infected are black persons	16	8.2	20	8.2	0.001	0.975

* *P* < 0.05

**Table 5.** Comparison of attitude and practices about COVID-19 among medical students in relation to academic grade

Variables	Preclinical (*N* = 194)		Clinical (*N* = 245)		*X* ^*2*^	*P*-Value
	*n*	%	*n*	%		
Do you believe that COVID-19 will finally be successfully controlled?					6.429	0.040*
Agree	134	69.1	148	60.4		
Disagree	20	10.3	46	18.8		
Don’t know	40	20.6	51	20.8		
Do you have confidence that Egypt can win the battle against the COVID-19 virus?					0.403	0.818
Agree	82	42.3	110	44.9		
Disagree	65	33.5	81	33.1		
Don’t know	47	24.2	54	22.0		
In recent days, have you gone to any crowded place?					0.192	0.664
Yes	47	24.2	55	22.4		
No	147	75.8	190	77.6		
In recent days, have you worn a mask when leaving home?					0.1824	0.177
Yes	117	60.3	132	53.9		
No	77	39.7	113	46.1		
In recent days, have you used disinfectants to clean your house?					0.101	0.750
Yes	185	95.4	232	94.7		
No	9	4.6	13	5.3		

* *P* < 0.05

### Assessment of Attitudes

Regarding a successful control of the COVID-19 pandemic, a majority of participants confirmed that it would be controlled (64.2%). Less than half of participants trusted that Egypt could win the fight against COVID-19 (43.7%), while almost one-third of the participants did not have a belief that Egypt can win the battle against the COVID-19 virus (33.3%). Almost a quarter of the participants did not know whether Egypt could win the battle or not (23%).w

### Assessment of Practices

Among all participants, 76.8% stated that they have not visited any crowded places recently. The majority of participants said that they were keen to wear facemasks whenever they were in public places (56.7%). Last, the majority of the participants reported cleaning their houses with disinfectants (94.8%).

Table 3 illustrates that females significantly have more confidence that Egypt will overcome the problem of COVID-19 (48.9%) than males (38.0%) (*P* = 0.005), and Table 4 shows the differences in knowledge in relation to academic grade were almost all not significant except for identification of fever as one of the symptoms and the fact that flu vaccine is not protective for COVID-19 which were reported significantly more by students of clinical grades (*P* = 0.014 and 0.03, respectively).

Table 5 explains attitude of pre-clinical students towards successful control of the disease was significantly more positive (69.1%) compared to clinical students (60.4%) (*P* = 0.040). Other items related to attitude and practices showed no significant difference in relation to academic grade of participants.

## Discussion

COVID-19 has postponed training of medical students in different universities due to the closure of campused during lockdown. During pandemics, such as COVID-19, the health-care system is put under great pressure, so much so that it forces authorities to recruit medical undergraduates to provide medical care to patients, exposing the students to the risk of infection.^13^ Moreover, medical students represent common references for health-care advice for family members and friends,^14,15^ particularly senior students (clinical stages).^16^ Thus, it is crucial to assess medical students’ knowledge, attitudes, and practices toward the novel coronavirus.

In the current study, the majority of undergraduates had an acceptable level of knowledge related to COVID-19. This coincides with Çalışkan et al., who assessed senior medical undergraduates’ knowledge regarding the COVID-19 pandemic in a Turkish university, and found that they had a moderate level of knowledge.^17^ Therefore, medical students need to be updated with medical information related to COVID-19 not only from research articles, but also from academic media and webinars.

Among the participants studied, female students were more knowledgeable about COVID-19 infection regarding main clinical symptoms (fever, fatigue) and high-risk groups (pregnant females, chronic patients). It coincides with Gao et al., who conducted a Web-based cross-sectional study among 588 medical and nonmedical students in China and found that female students had a better conception regarding portals of transmitting the disease and how to prevent the spread of coronavirus than male students.^18^ Female students were more interested in following the updated information presented in well-trusted platforms. In addition, the issue of pregnancy can be of more concern for females than males. Moreover, medical students in the clinical stages had more knowledge regarding coronavirus than those in the preclinical stages. This is in accordance with a study among Iranian medical students reporting that intern students were significantly more knowledgeable than younger students.^19^ This difference can be illustrated by the fact that senior students have more confidence and skills that enable them to interrelate with different scientific research and give more better interpretation of information.

The majority of preclinical students had the belief that the current pandemic will be controlled. This confidence was low regarding the ability of Egypt to control the disease. Moreover, female students were more optimistic regarding Egypt’ success in controlling the current situation than males. This was contrary to the results of a Turkish study reporting that one-third of Turkish final year medical undergraduates trusted that the current pandemic could not be controlled.^17^ Despite the wide sharing of epidemic situation statistics by the Ministry of Health and professional and public communities, confidence of students in the ability of Egypt to control the disease was less than half of them. This refers to the lack of confidence of students in available regulations and resources needed to control the epidemic in Egypt. Decision-makers and medical authorities in Egypt should exert more efforts and show more transparency in sharing information with medical professionals to gain their confidence and cooperation in dealing with this crisis.

In the current study, most of the participants adopted precautionary measures such as using disinfectants in cleaning issues, avoiding crowded places, and wearing a facemask. On the contrary, a study among students in 6 medical schools in Jordan, 61% of undergraduates never used the face-masks.^13^ Only 30.3% of medical students and university staff in a Kazakhstani university reported that it was difficult to maintain social distance isolation.^20^ This could be due to differences in rules issued by governments and previous experiences in dealing with other pandemics. Also, it emphasizes the necessity of raising awareness of students about face-mask practices as advised by health authority all over the world.

The Egyptian Ministry of Health announced from May 2020 the necessity of wearing a facemask as a preventive measure through different channels. In a study among Egyptians in March 2020, results showed that three-quarters of the participants believed that protection of infection could be achieved through putting on facemasks.^21^ It indicates that Egyptians are aware of necessary precautions but their commitment to use protective measures is not as strong. Hence, more health education regarding the need for using protective measures and wearing a face-mask is essential, especially among medical students.

## Conclusions

Egyptian medical students had an acceptable level of knowledge, positive attitude, and good practices of preventive measures regarding the COVID-19 virus. There is no significant difference in almost all items of knowledge, attitude, and practices in relation to gender or academic grade.

### Recommendations

It is crucial to design strategies to raise the awareness and knowledge of medical students about public health disasters and medical emergencies. Assessing knowledge and different precautionary measures to contain the disease represents pivotal steps in determining the future efforts toward the educational process.

### Limitations of Study

There are several limitations regarding the current study. First, the attitude and practice sections consisted of only 5 items and can be modified in future studies. Second, the current study included only students from a governmental medical institution. Future researchers should consider recruiting an equal number of students from both government and private institutions for better understanding of their knowledge and perceptions. In addition, recall bias could result as collection of data depended on students’ memory abilities. Moreover, we used a Web-based survey method in the study, so we expected selection bias. Finally, the participants had access to the Internet for their computers and cellphones; thus, participants may have higher income or better educational access than those who did not have similar facilities.

### Future Work

These findings shed more light on the importance of public health preparedness by implementing awareness educational programs for medical students about the current pandemic. This will promote their knowledge and inculcate positive perception. In addition, medical students act as role models for all people in the community. Hence, such surveys will be instrumental in quantifying the gaps in knowledge. Therefore, it is crucial to target improving orientation of students as the rest of the world prepares to resume medical training, an essential intervention for the continuity of essential medical services, especially in low resource settings.
